# Feeling Asian Together: Coping With #COVIDRacism on Subtle Asian Traits

**DOI:** 10.1177/2056305120948223

**Published:** 2020-07-30

**Authors:** Crystal Abidin, Jing Zeng

**Affiliations:** 1 Curtin University, Australia; 2 University of Zurich, Switzerland

**Keywords:** COVID-19, anti-Asian racism, discursive activism, Subtle Asian Traits, Facebook groups

## Abstract

Since the onset of COVID-19, incidents of racism and xenophobia have been occurring globally, especially toward people of East Asian appearance and descent. In response, this article investigates how an online Asian community has utilized social media to engage in cathartic expressions, mutual care, and discursive activism amid the rise of anti-Asian racism and xenophobia during COVID-19. Specifically, we focus on the 1.7-million-strong Facebook group “Subtle Asian Traits” (SAT). Throughout the COVID-19 pandemic, the 1,200 new posts it publishes daily have swiftly pivoted to the everyday lived experiences of (diaspora) East Asians around the world. In this article, we reflect on our experiences as East Asian diaspora members on SAT and share our observations of meaning-making, identity-making, and community-making as East Asians collectively coping with COVID-19 aggression between January and May 2020.

## Subtle Asian Traits: A Repository of Being Asian

It’s fucking me up emotionally how one day a Korean film with subtitles can sweep The Academy Awards and the idea of inclusivity/progression is in the air. A month and a half later, I’m being told I eat bats and Asian American hate-crimes are on the rise. (28 March 2020)

This quote—posted by a member of Subtle Asian Traits (SAT), a Facebook group—exemplifies how the ongoing health crisis has shaped what it means and feels to be Asian. As two Asian diaspora scholars who have spent several years living in a string of cities where our cultural identities have been questioned, contested, and commodified, a Facebook group like SAT can feel like a mothership to soothe returning aliens to an online “home.” While popular among East Asians in general, SAT caters especially to the Asian diaspora, having been launched in 2018 “as a joke” by eight Asian-Australian teenagers to reflect on their intersectional experience. Since then, SAT boasts an impressive 1.7 million members, including actors, musicians, and public figures of Asian descent who praise it for championing Asian representation through its leadership and advocacy work online and offline.

On SAT, everyday experiences of “being Asian” are parsed through banal or critical internet vernacular as reminders and reflections of our cultural identities: members celebrate memes, viral videos, and Asian excellence alongside sincere diary entries, heart-breaking sob stories, and riveting confessions about “growing up Asian.” Members congregate daily to share their origin stories, family cultures, food preferences and traditions, stereotypes about Asian bodies, and entertainment. In internet speak, SAT is tl;dr a repository of a universal (East) Asian experience and a validation of one’s diaspora struggles, all packaged into a massive Facebook group.

## Coping With the Pandemic, the Subtle Asian Way

In the age of COVID-19, SAT has become a congregational node for the Asian diaspora community on Facebook. From propagating “quarantine trends” (e.g., homemade dalgona coffees which require the effort of whipped coffee and milk but are Instaworthy to simulate the café experience; and recommendations of Korean dramas in every genre to soothe the soul) to joking about Asian mothers’ pseudo-scientific anti-COVID remedies, SAT’s 1000+ daily posts from its members have swiftly pivoted to reflecting on what it means to be “Asian” during the pandemic. However, SAT is not just about memes and humor. (For the record, the authors’ favorite meme was when SAT members collectively agreed that all our Asian mothers were graduating from the “WhatsApp and WeChat University of Misinformation”; someone even designed a mock graduation certificate for our moms.)

**Figure 1.**
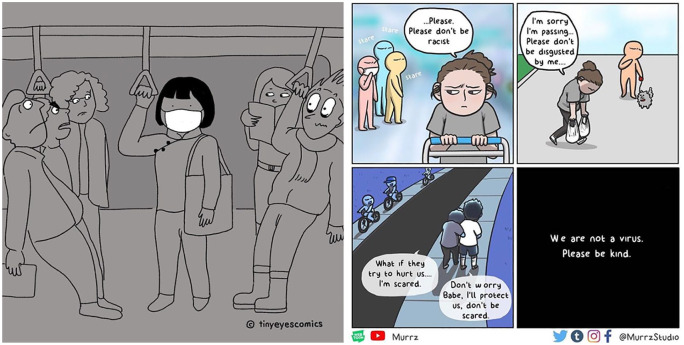
Examples of popular images circulated in SAT depicting experiences of racism.

Since knowledge of the outbreak first occurred in early 2020, disheartening incidents from people of East Asian appearance have been reported worldwide (Giuffrida & Willsher, 2020) and on SAT (Figure 1), recounting experiences of being verbally and physically attacked. In the fight against #COVIDRacism, social media serves as the key arena where Asians can speak out about their own encounters and launch various counter campaigns (Zeng, 2020). In particular on SAT, the pandemic has shifted the tonality of camaraderie and community to focus on sharing, resolving, and teasing out the other universal East Asian experience of coping with and surviving COVID-19 race-based aggression.

In our study of SAT’s comments sections, we have observed various forms of coping strategies used by group members to counteract racism, most of which are expressed through lengthy threads. In response to such shared struggles, as participant-researchers we observed that SAT members collectively cope with the rising prejudice against Asians through Catharsis, Escalation, and Problem solving.

**Figure 2.**
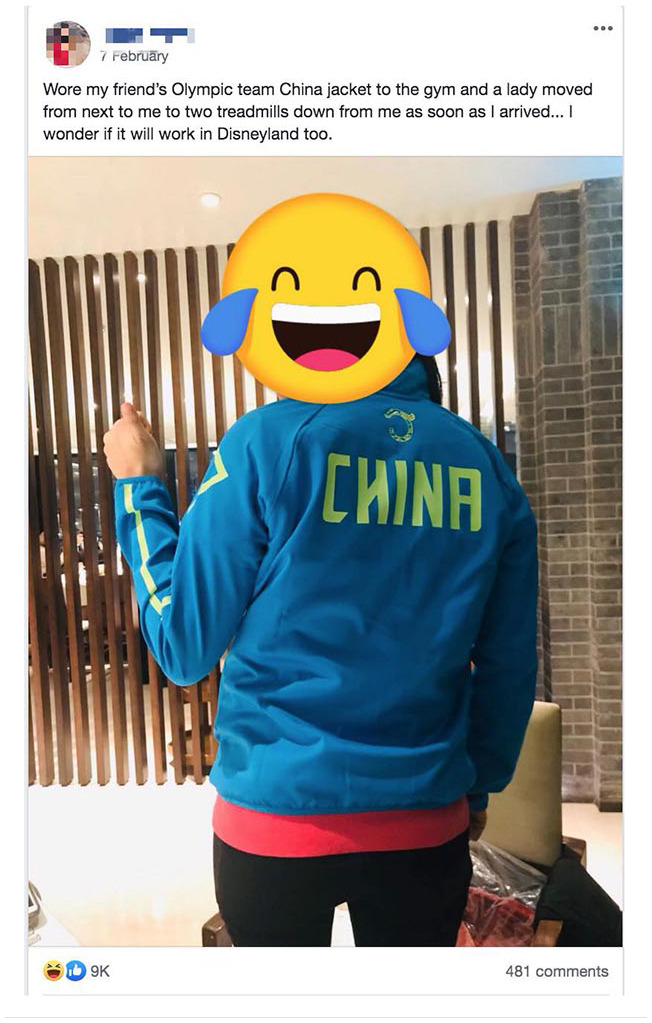
Screenshot of an SAT post joking about being avoided in public.

Amid the pandemic of #COVIDRacism, SAT has become the “go to” arena for airing our grievances and for seeking resonance and support within the East Asian (diaspora) community. We describe this coping strategy where users share their own feelings and experiences as *catharsis*. For instance, SAT members often circulate self-deprecating TikTok videos of themselves holding in coughs out of fear of being ostracized, or humorous stories of how our Asian appearances command spatial buffers when we are in public spaces (Figure 2).

**Figure 3.**
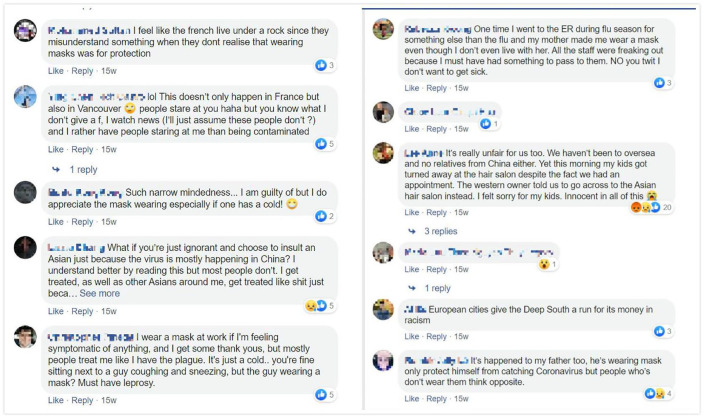
Screenshots of SAT members’ discussions about wearing masks.

“Mask on vs mask off” was another hot topic in the group during the early phase of the outbreak. On one hand, for most Asian people, wearing masks is a common and natural thing to do in crowded public places, especially when one is ill, when dealing with bad environmental conditions (e.g., fine dust in South Korea, pollution in China), to keep warm (e.g., countries with winter seasons), or even to retain a degree of anonymity or privacy (also influenced by East Asian celebrity cultures where masks are worn when makeup is off). On the other hand, as diaspora members in countries without this cultural context, we are concerned about cultural clashes, misunderstandings, and unintended consequences around mask wearing. SAT members actively shared our own stories about Asians wearing masks in Global Northern countries receiving eyerolls, being avoided, or even being attacked in public (Figure 3).

Escalation is the second strategy SAT members have commonly utilized to cope with #COVIDRacism and refers to the practice of members amplifying the reach and visibility of anti-Asian racism incidents. In cases of assault, SAT members often suggest that we make reports to the authorities, media, and other Facebook support groups (e.g., Crimes Against Asians). When confronted with racist assaults, it seems to be a cultural response for early-generation diaspora Asians to take non-confrontational approaches such as “quietly enduring the pain” or “walking away”—indeed many of our comments threads referenced these notions in a variety of East Asian adages and idioms. This norm of “not making a scene” has made Asians the invisible victims of racism (Chou & Feagin, 2015). But unlike our parents’ generation, the younger Asian diaspora on SAT are more vocal and more willing to decry the unfair treatment they receive, and strategize with others on possible solutions.

From providing advice on self-defense to offering tangible help to Asian businesses suffering from the lack of customers, SAT members also provide *problem-solving* solutions. Providing advice and comforting messages to each other regarding our life and career struggles has always been a recurring theme of posts and comments in this group (this shared commiseration is partly in jest and partly genuine emotional support for those of us who have grown up with #HighExpectationAsianDads and #TigerMoms). Throughout the Pandemic, SAT’s “Agony aunts” have similarly offered suggestions on some measures that fellow Asians can take when reacting to COVID-triggered mistreatment. For example, circulated footage of an Asian couple getting beaten up on a subway in Philadelphia triggered widespread outrage and heated discussion, and the group’s discussions called upon fellow Asians to intervene and take action if we ever witnessed such a situation.

## The Limitations of Subtle Asian Commiseration

The centrality of SAT as a hub for the East Asian diaspora to cope with racism is evident through the volume of content posted daily. However, this community is by no means a virtual utopia with only “rainbows and butterflies” (or “kpop and boba,” in the vernacular of our fellow SAT members). Throughout the pandemic, incidents uncovering in/out-group conflicts have regularly emerged in the group. In January and February, when COVID-19 first emerged in Europe and the United States, many of the responses from non-Chinese SAT members were in the form of complaints that they were “not even Chinese” and should not be subject to mistreatment. This form of rhetoric incurred the wrath of other commenters who suggested that unity is important as racists do not care for such subtleties. In early March, when footage of anti-Asian assaults circulated on SAT, the comments section on SAT was also flooded with racist comments alluding to the African-American identity of the aggressor. There were yet other threads where inter-Asian and inter-minority tensions broke out, in displays of “competitive grief olympics” or “competitive onedownmanship” (Ask & Abidin, 2018) where users attempted to measure and compare the extent of their pain and outrank each other.

On threads where aggressive comments accumulated, trolls emerged, or conversations derailed from the ethos of SAT (e.g., offering political commentary on actions by politicians and authorities, addressing racist and xenophobic language), admins and moderators would step in to lock the comments and curtain further discussion. While it was found in other studies of antagonistic comments sections that disagreements can be used as springboards for negotiations and teachable moments (Abidin, 2019; Johnston, 2017; Uldam & Askanius, 2013), this was not usually the case for SAT given the truncation of conversations due to admin blocks. Furthermore, due to the volume of posts and pace of new updates on the page in a non-chronological fashion, it is unlikely that members would return to previously seen posts to keep track of the development of discourse on comment threads, unless they had specifically bookmarked them to do so. Although these occasional incidents of “fighting racism with racism” and “out-grieving each other” are significantly outweighed by numerous voices calling for rationality and solidarity, they still reveal the limitations of SAT as a space for discursive activism given that the values and ethos of all 1.7 million of us cannot be singularly aligned.

## Subtle Asian Scrolling for Companionship

As the weeks turned into months, the daily routine of scrolling through SAT has become a way of equipping ourselves with vocabularies, literacies, and potential reactions for managing the fast-changing COVID-19 situation. Like many members of SAT, we would sift through the pages and send each other links to “must see” posts, often closely monitoring the ones that we felt were on the cusp of “going viral” (Jing’s attempt at dalgona coffee was a moderate success whereas Crystal’s homemade bubble tea never eventuated). Other times, we had tagged other diaspora friends in posts that related to recent accounts of their own experiences with anti-Asian racism and xenophobia, especially if these had occurred in cities in the vicinity of their residence.

Although there are often encouraging advocacy and activism initiatives among SAT members—such as the grassroots organization of small relief and aid for Asians businesses that were the targets of racially motivated attacks, tips and hacks for educating elderly family members about COVID-19 misinformation, and suggestions for maintaining personal safety and well-being—we do not want to overstate the singularity or utility of SAT. Ultimately, the imagined utopia and harmony of a space like SAT is fragile and superficial and can be easily disturbed or destabilized when the tonality of topics ventures into more serious discussions. This was acknowledged by the SAT admins in a formal group announcement:

[. . .] However, please also recognize that SAT is currently run by a group of mostly volunteer college students and generally focuses on sharing light-hearted posts—so the group might not currently be equipped to ensure the kind of nuanced, civil discussion platform around certain topics in our community [. . .]. (2 March 2020)

For the most part, what SAT has really provided for members during COVID-19 is a sense of companionship through the ups and downs: We have shared heart-wrenching anecdotes detailing experiences with racism, missing the funerals of loved ones far away, and precariousness of job security; alongside the first world problems of missing constant access to bubble tea, deteriorating beauty standards, and running out of Korean dramas to watch.

Alongside its role as an evolving repository of “How To Be (East) Asian,” through the sharing of family histories, traumas, material consumption, and niche Asian pop cultural references, SAT is also a living archive of How It Feels To Be (East) Asian in the time of COVID-19. The impressions of normalcy, fun, and the semblance of unity through laughing together is a welcome albeit brief distraction, from the looming crisis outside of our screens and beyond our control.
